# In vitro cytotoxicity of Auger electron-emitting [^67^Ga]Ga-trastuzumab

**DOI:** 10.1016/j.nucmedbio.2019.12.004

**Published:** 2019-12-13

**Authors:** Muhamad Faiz bin Othman, Elise Verger, Ines Costa, Meena Tanapirakgul, Margaret S. Cooper, Cinzia Imberti, Valerie J. Lewington, Philip J. Blower, Samantha Y.A. Terry

**Affiliations:** a Department of Imaging Chemistry and Biology, School of Biomedical Engineering and Imaging Sciences, St. Thomas' Hospital, London, SE1 7EH, United Kingdom; b Guy's & St Thomas' NHS Foundation Trust, Kings College London, London SE1 9RT, UK

**Keywords:** Gallium-67, Molecular radiotherapy, Auger electrons, Tris(hydroxypyridinone), Radiobiology, Trastuzumab

## Abstract

**Introduction:**

Molecular radiotherapy exploiting short-range Auger electron-emitting radionuclides has potential for targeted cancer treatment and, in particular, is an attractive option for managing micrometastatic disease. Here, an approach using chelator-trastuzumab conjugates to target radioactivity to breast cancer cells was evaluated as a proof-of-concept to assess the suitability of ^67^Ga as a therapeutic radionuclide.

**Methods:**

THP-trastuzumab and DOTA-trastuzumab were synthesised and radiolabelled with Auger electron-emitters ^67^Ga and ^111^In, respectively. Radiopharmaceuticals were tested for HER2-specific binding and internalisation, and their effects on viability (dye exclusion) and clonogenicity of HER2-positive HCC1954 and HER2–negative MDA-MB-231 cell lines was measured. Labelled cell populations were studied by microautoradiography.

**Results:**

Labelling efficiencies for [^67^Ga]Ga-THP-trastuzumab and [^111^In]In-DOTA-trastuzumab were 90% and 98%, respectively, giving specific activities 0.52 ± 0.16 and 0.61 ± 0.11 MBq/μg (78–92 GBq/μmol). At 4 nM total antibody concentration and 200 × 10^3^ cells/mL, [^67^Ga]Ga-THP-trastuzumab showed higher percentage of cell association (10.7 ± 1.3%) than [^111^In]In-DOTA-trastuzumab (6.2 ± 1.6%; *p* = 0.01). The proportion of bound activity that was internalised did not differ significantly for the two tracers (62.1 ± 1.4% and 60.8 ± 15.5%, respectively). At 100 nM, percentage cell binding of both radiopharmaceuticals was greatly reduced compared to 4 nM and did not differ significantly between the two (1.2 ± 1.0% [^67^Ga]Ga-THP-trastuzumab and 0.8 ± 0.9% for [^111^In]In-DOTA-trastuzumab). Viability and clonogenicity of HER2-positive cells decreased when each radionuclide was incorporated into cells by conjugation with trastuzumab, but not when the same level of radioactivity was confined to the medium by omitting the antibody conjugation, suggesting that ^67^Ga needs to be cell-bound or internalised for a therapeutic effect. Microautoradiography showed that radioactivity bound to individual cells varied considerably within the population.

**Conclusions:**

[^67^Ga]Ga-THP-trastuzumab reduced cell viability and clonogenicity only when cell-bound, suggesting ^67^Ga holds promise as a therapeutic radionuclide as part of a targeted radiopharmaceutical. The causes and consequences of non-homogeneous uptake among the cell population should be explored.

## 1 Introduction

With regulatory approval of beta particle-emitting radiopharmaceuticals such as [^177^Lu]Lu-DOTATATE [1], early successes in trials of alpha particle-emitting therapy ^223^Ra [2] and an increasing choice of therapeutic radionuclides, chelators and radiochemistry techniques to choose from, this is an exciting time for molecular radiotherapy. Examples of therapeutic radiopharmaceuticals in routine clinical use include ^131^I for thyroid cancer, [^131^I]mIBG for neuroblastoma, ^90^Y- and ^177^Lu-radiopeptides for neuroendocrine tumours, and ^90^Y-labelled microspheres (albeit defined for regulatory purposes as a medical device rather than a radiopharmaceutical) for hepatic tumours [3,4]. Most prominent among new beta particle-emitting radiopharmaceuticals undergoing clinical trials is [^177^Lu]Lu-PSMA [5]. The potential of high linear energy transfer alpha particle-emitters, such as ^213^Bi, ^225^Ac, ^211^At, and ^227^Th [6–8] to treat low volume tumours effectively while limiting healthy tissue toxicity is being explored. By comparison with alpha particles (<100 μm particle range), the short range (often <1 μm) of Auger electrons and related secondary electrons would be expected to increase the therapeutic ratio and further mitigate non-target toxicity [9]. Although likely to be less effective than beta emitters in large tumours because of the lack of “cross-fire” contribution to the tumour radiation dose, Auger electron-emitters could be effective against single cells and micrometastases which are ineffectively treated by beta-emitters [10] and are responsible for recurrence of several tumour types [11,12].

Among therapeutic Auger electron-emitters, ^111^In has so far been closest to clinical translation and has been evaluated in both breast cancer [13] and neuroendocrine tumours [14]. ^67^Ga, which has been used clinically for SPECT imaging of inflammation and infection [15,16] also emits Auger electrons but its use as a therapeutic radionuclide is relatively unexplored [17–22]. Despite promising in vitro results, including its use in a radiopharmaceutical targeted towards the CD74 receptor on human B-lymphoma cells [21,22], low specific activities and a lack of purpose-designed gallium chelators have hindered progress with ^67^Ga therapy. The recent development of several new chelators [23–30], including the tris(hydroxypyridinone) chelators, allow kit-based radiolabelling of biomolecules, including proteins, with gallium radionuclides at high specific activities [31–33]. This development provides renewed opportunities for clinical translation to assess the tumouricidal potential of ^67^Ga preclinically and clinically as a radionuclide for molecular radionuclide therapy. Our recent studies have shown that ^67^Ga caused more DNA damage per Bq than ^111^In in a cell-free system. The cellular radioactivity required to kill 50% and 90% of HCC1954 breast cancer cells was 6- and 1.5-fold less, respectively, for ^67^Ga than for ^111^In [9,34]. Lower plasmid DNA damage was caused by ^67^Ga than ^111^In when the radionuclides were physically separated from the DNA, suggesting that ^67^Ga would cause less unwanted damage to surrounding non-target cells than ^111^In [34]. These data suggest that ^67^Ga could be selectively cytotoxic if targeted to tumour cells, with minimal damage to nearby non-targeted cells.

A widely explored tumour-specific molecular target is HER2, a receptor expressed on breast cancer cells. The antibody trastuzumab binds to HER2 and so is a potential basis for targeted delivery of radionuclides and evaluation of the therapeutic potential of ^67^Ga. Even in patients with primary tumours classified as HER2-negative, their metastases can still prove HER2-positive [35]. HER2-positivity in patients with breast cancer is associated with aggressive disease, poor prognosis and shortened overall survival [36]. A combination of trastuzumab, pertuzumab and docetaxel was able to partially overcome trastuzumab resistance and extend survival of patients with metastatic HER2-positive breast cancer from 15 (trastuzumab alone) to 56.5 months [37]. This drug combination is now used as first-line therapy for metastatic HER2-positive breast cancer. Another successful approach uses antibody-drug conjugates, such as the trastuzumab conjugate T-DM1, which delivers a toxic payload of emtansine to HER2-positive breast cancer cells [38]. The success of T-DM1 suggests that the efficacy of using a radionuclide, such as an Auger electron-emitter, as the toxic payload, should be explored [38]. In this study, trastuzumab was used to target ^67^Ga to HER2-positive breast cancer cells to determine the ability of ^67^Ga to kill these cells selectively compared to [^111^In]In-DOTA-trastuzumab.

## 2 Materials and methods

### 2.1 Radionuclide preparation

Radiolabelling was carried out with ^67^Ga used either as supplied ([^67^Ga]Ga-citrate at a concentration of 80–160 MBq in 2.2 mL; Mallinckrodt, Netherlands) or after converting to [^67^Ga]GaCl_3_ as previously described [39]. For conversion to [^67^Ga]GaCl_3_, [^67^Ga]Ga-citrate was diluted to 5 mL with distilled water and concentrated on a SEP-PAK silica light 120 mg column (Waters, USA) at 1 mL/min. Trapped ^67^Ga was eluted with 0.1 M HCl in 50 μL fractions. The radioactivity concentration in the highest fraction (usually fraction 6 or 7) was 1010 ± 388 MBq/mL [^111^In]InCl_3_ (110–200 MBq) was used as supplied in 0.3 mL hydrochloric acid (Mallinckrodt, Netherlands).

### 2.2 Chelator conjugation to trastuzumab

Trastuzumab was conjugated to chelators tris(hydroxypyridinone) (H_3_-THP-Ph-SCN; THP, synthesised as previously described [40]) and S-2-(4-isothiocyanatobenzyl)-1,4,7,10-tetraazacyclododecane tetraacetic acid (*p*-SCN-Bn-DOTA, Macrocyclics, USA; DOTA) for radiolabelling with ^67^Ga and ^111^In, respectively.

#### 2.2.1 THP-trastuzumab

Trastuzumab (435 μL, 22.96 mg/mL) in physiological saline was incubated with 50 mM ethylenediaminetetraacetate sodium (EDTA) solution for 30 min to remove any adventitious metal ions. The mixture was filtered at 4000 g in a Vivaspin 2 filter (Sartorius, Germany). The filter was washed with metal-free HEPES and the antibody thus recovered had a concentration of 12.46 ± 0.05 mg/mL (as measured by NanoDrop spectrophotometer) in metal-free HEPES. This solution of trastuzumab (3.23 mg, 260 μL, pH 8.7) was incubated with THP (21 μL of 10 mM solution in DMSO) at 10:1 chelator:antibody molar ratio at room temperature (2 h) and then 4 °C (2 h). The mixture was applied to a Sephadex PD MidiTrap G-25 size exclusion column (GE Healthcare) and eluted with 0.2 M ammonium acetate. The fraction eluting between 1 mL and 2.5 mL was applied to a Sephadex PD-10 size exclusion column (GE Healthcare), and eluted with 0.2 M ammonium acetate solution in 500 μL fractions. Fraction 8 (protein concentration 3.5 mg/mL, measured by NanoDrop) was used for radiolabelling.

#### 2.2.2 DOTA-trastuzumab

850 μL trastuzumab (23.5 mg/mL in saline) was dialysed overnight in a 3.5 kDa MWCO Slide-a-lyzer™ cassette (ThermoFisher, USA) against 0.2 M, pH 5.5 sodium acetate containing 2 g/L chelex (Sigma, UK). DOTA conjugation to trastuzumab (10 mg, 12.8 mg/mL) was achieved by incubating a reaction solution containing a 40:1 M ratio of chelator to antibody, i.e. 1.1 mg DOTA (*p*-SCN-Bn-DOTA, Macrocyclics, USA; 110 mg/mL in 0.25 M pH 5.5 sodium acetate) to which 83 μL 1 M NaHCO_3_, pH 9.5 was added and the mixture stirred for 60 min at room temperature. The conjugated antibody was dialysed against 0.2 M, pH 5.5 ammonium acetate containing 2 g/L chelex to remove unbound p-SCN-Bn-DOTA, furnishing a 13.5 mg/mL DOTA-trastuzumab solution (measured by NanoDrop).

### 2.3 Radiolabelling and quality control

#### 2.3.1 [^67^Ga]Ga-THP-trastuzumab

[^67^Ga]Ga-citrate (48.6 MBq/mL) was added as aliquots of 25 μL (1.2 MBq), 100 μL (4.9 MBq) and 200 μL (9.7 MBq) to 100 μg THP-trastuzumab in 0.2 M ammonium acetate (3.5 mg/mL). pH was adjusted to 6.5 with 0.1 M NaHCO_3_. The mixture was left for 15 min at room temperature. Unchelated [^67^Ga was scavenged with 2 μL 50 mM EDTA. Alternatively, [^67^Ga]GaCl_3_ (58.3 ± 11.9 MBq in 80 μL) was added to 100 μg THP-trastuzumab in 0.2 M ammonium acetate (3.5 mg/mL), pH was adjusted to 6.5 with 0.1 M NaHCO_3_ and the mixture was left for 15 min at room temperature.

#### 2.3.2 [^111^In]In-DOTA-trastuzumab

[^111^In]InCl_3_ (94.1 ± 29.4 MBq in 120 ± 30 μL) was mixed with 250–430 μL 0.1 M ammonium acetate, pH 5.5, and added to 150 μg DOTA-trastuzumab. The mixture was kept at 40 °C for 2 h. Unchelated ^111^In was scavenged with 2 μL of 50 mM EDTA.

#### 2.3.3 Quality control

Labelling efficiencies were determined by paper chromatography on Whatman 1 paper (1 cm × 6.5 cm) with a mobile phase of 1 mM DTPA and were almost always >95%. Preparations in which the radiochemical purity was ≤95% were purified using a PD-10 column in 1 mL fractions of PBS containing 0.1% BSA.

### 2.4 Cell culture

MDA-MB-231 (HER2-negative) and HCC1954 (HER2-positive) breast cancer cell lines were grown as monolayers at 37 °C in a humidified atmosphere with 5% CO_2_ in high glucose (4.5 g/L) Dulbecco's Modified Eagle Medium (DMEM; PAA Laboratories) and RPMI-1640, respectively. Media were supplemented with 1.5 mM l-glutamine (PAA Laboratories, Austria), 10% foetal bovine serum (Invitrogen) and penicillin/streptomycin (Invitrogen) to create full medium. Cells were harvested using trypsin-EDTA for experimental use.

### 2.5 Binding assay

5 × 10^4^ HCC1954 cells per well were seeded in a 6-well plate 24 h prior to the experiment and incubated in a 5% CO_2_ incubator at 37 °C. 4 nM [^67^Ga]Ga-THP-trastuzumab or [^111^In]In-DOTA-trastuzumab (0.08 and 0.09 MBq, respectively) were added to cells in a final volume of 250 μL full medium followed by incubation for 1 h at 37 °C. Medium was collected and combined with supernatant from two subsequent PBS wash steps to measure unbound activity. Cells were lysed in their wells with 1 mL 0.1 M NaOH and the solutions transferred to counting tubes to measure bound activity. Radioactivity was measured with a Wallac 1282 Compugamma gamma counter (Wallac, Germany) to calculate the percentage of cell-bound radioactivity. Controls included non-internalised activity (antibody-free [^67^Ga]Ga-THP and [^111^In]In-DOTA; 0.5 MBq) in HCC1954 cells, and HER2-negative MDA-MB-231 cells incubated with 4 nM [^67^Ga]Ga-THP-trastuzumab or [^111^In]In-DOTA-trastuzumab.

### 2.6 Internalisation assay

1 × 10^6^ HCC1954 cells in suspension were incubated with 4 nM [^67^Ga]Ga-THP-trastuzumab or [^111^In]In-DOTA-trastuzumab (0.08 and 0.09 MBq, respectively) in 1 mL medium for 1 h at 37 °C. Unbound activity in the medium was collected and combined with washings from two PBS wash steps after centrifuging at 1500 rpm for 5 min. Cell pellets were subjected to an acid wash by resuspending in 1 mL 500 mM NaCl and 200 mM NaOAc (pH 2.5) for 5 min at 4 °C [41]. Cells were then centrifuged to separate membrane-associated activity (supernatant) from internalised activity (pellet). Radioactivity was measured as above.

### 2.7 Viability

[^67^Ga]Ga-THP-trastuzumab or [^111^In]In-DOTA-trastuzumab (0.18 MBq/μg, 27 GBq/μmol) was added to 50 × 10^3^ HCC1954 cells in 250 μL full medium per well at 0, 4, 10, 40 and 100 nM, i.e. 0, 0.02, 0.06, 0.2 and 0.6 MBq, respectively, per well. Controls included untreated HCC1954 cells, HCC1954 cells incubated with 100 nM non-radiolabelled trastuzumab, non-cell-binding activity ([^67^Ga]Ga-THP and [^111^In]In-DOTA; 0.5 MBq), and MDA-MB-231 cells incubated with 100 nM [^67^Ga]Ga-THP-trastuzumab or [^111^In]In-DOTA-trastuzumab. Following a one-hour incubation, the supernatant was removed and cells were washed in PBS and resuspended in 2 mL full medium. Viability was measured after 3 days incubation in the 5% CO_2_ incubator at 37 °C using the trypan blue exclusion assay. Viability values were normalised to the untreated group (set at 100%).

### 2.8 Clonogenic assay

2 × 10^3^ cells per well were plated in 6-well plates 24 h prior to the experiment. HCC1954 cells were incubated with 0.18 MBq/μg (27 GBq/μmol) radiolabelled trastuzumab in 500 μL for 1 h at 37 °C. The radioactive supernatant was then removed and cells were incubated in 2 mL full medium in a 5% CO_2_ incubator at 37 °C. The medium was changed every 3 days. After 9–14 days, cells were fixed and stained with 1:1 methanol/1% crystal violet (Sigma, UK). Surviving fractions were calculated by counting colonies consisting of >50 cells. Controls included HCC1954 cells incubated with 100 nM non-radiolabelled trastuzumab, non-cell-binding radiopharmaceutical ([^67^Ga]Ga-THP and [^111^In]In-DOTA; at 1 MBq) and MDA-MB-231 cells incubated with 100 nM [^67^Ga]Ga-THP-trastuzumab or [^111^In]In-DOTA-trastuzumab. Clonogenic survival values were normalised to the untreated group (set at 1.0).

### 2.9 Microautoradiography

1 × 10^6^ HCC1954 cells in suspension were treated with 0 or 4 nM [^67^Ga]Ga-THP-trastuzumab (0 and 0.04 MBq, respectively; 0.07 MBq/μg, 11 GBq/μmol) in 1 mL full medium. Preparations were incubated in a 5% CO_2_ incubator at 37 °C for 1 h and washed in PBS, fixed in 4% formaldehyde in PBS for 20 min at room temperature, washed and resuspended in 200 μL PBS. Gelatine was dissolved in PBS in a microwave oven. Once cooled to about 37 °C, 400 μL of the 18% gelatin solution was added to cells (200 μL PBS) in a 1 cm × 1 cm mould and kept at 4 °C for 15 min. Once solidified, the gelatine block was trimmed to into cubes of 4 × 4 × 4 mm, which were snap frozen in isopentane in liquid nitrogen. Sections 10 μm thick were cut in a cryotome (Bright Instrument Company Ltd., UK) and placed on positively-charged polylysine-coated slides. In a dark room, the slides were coated by dipping in K2 Ilford photographic emulsion (Ilford, Germany), which was prepared by adding 10 mL emulsion to 16.7 mL distilled water containing 0.01% glycerol and gently heating to 37 °C until dissolved. Dipped slides were left to dry horizontally at 4 °C for 24 h in a light-protected case, and developed at room temperature for 5 min in Kodak D19 (Kodak, USA; 15.8% (w/v) in distilled water). Slides were then dipped in 1% acetic acid for 30 s and then in 0.5% gelatin and dried at room temperature. Finally, autoradiographs were fixed in 30% sodium thiosulfate for 5 min, washed for 10 min and left to dry. Prior to imaging, 50 μL DAPI-containing mounting solution was applied to the slide and a coverslip was placed on top. Images were acquired with an EVOS FL Cell imaging microscope (Life Technologies, USA) and analysed with Image J (NIH, USA). The total number of silver grains around each cell was measured (within a circle 25 μm in diameter around the cell centre) in cells identified as DAPI-positive. The amount of signal in cells was corrected for background in an equivalent cell-free area of the image.

### 2.10 Statistical analysis

**Fig. 1:**
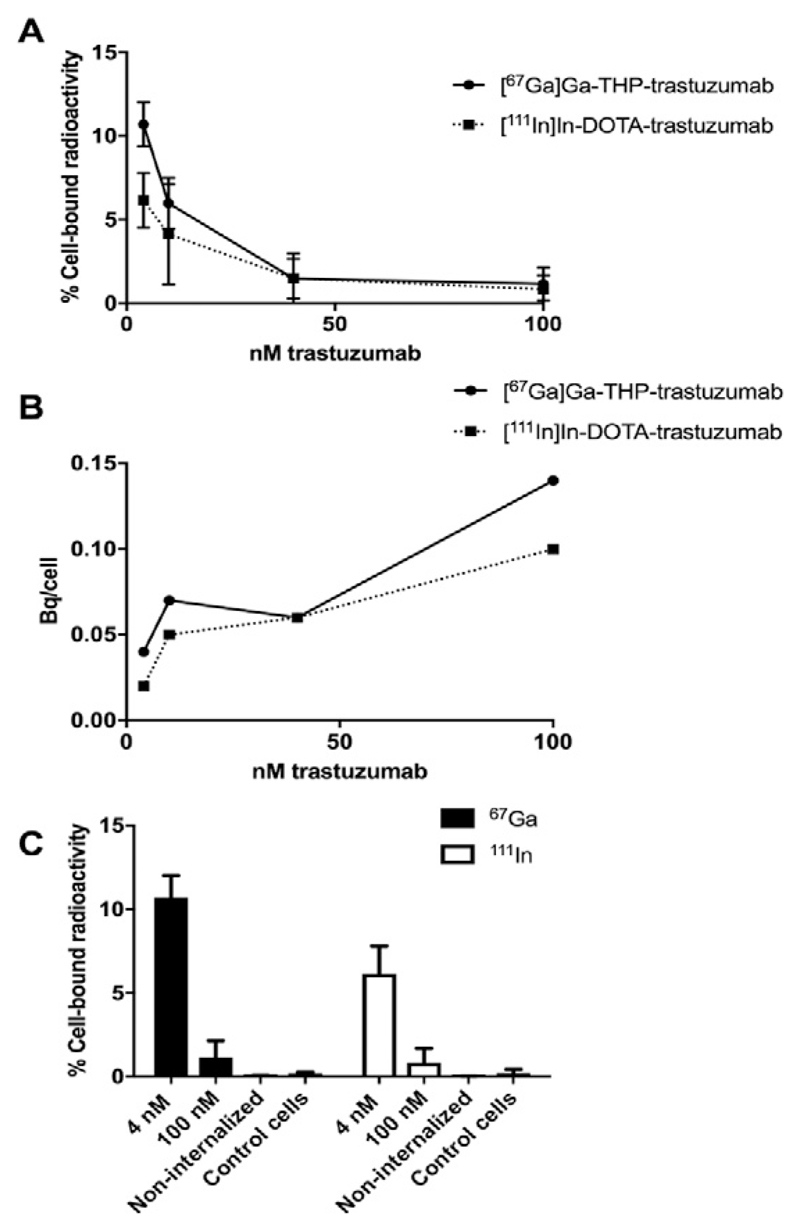
A. Binding of [^67^Ga]Ga-THP-trastuzumab and [^111^In]In-DOTA-trastuzumab to 50 × 10^3^ HER2-positive HCC1954 cells at increasing trastuzumab concentrations (n = 3). B. Calculated mean activity per HCC1954 HER2-positive cell (Bq/cell) for [^67^Ga]Ga-THP-trastuzumab and [^111^In]In-DOTA-trastuzumab. C. Binding of [^67^Ga]Ga-THP-trastuzumab and [^111^In]In-DOTA-trastuzumab to HCC1954 cells at 4 and 100 nM. Controls include non-internalised activity ([^67^Ga]Ga-THP and [^111^In]In-DOTA; 0.5 MBq) and HER2-negative MDA-MB-231 incubated with 4 nM [^67^Ga]Ga-THP-trastuzumab and [^111^In]In-DOTA-trastuzumab (control cells) (n = 3).

Statistical analysis was performed using Graphpad Prism Version 7.0c. An unpaired two-tailed *t*-test was used to analyse binding assay results comparing [^67^Ga]Ga-THP-trastuzumab uptake with [^111^In]In-DOTA-trastuzumab. A one-sample two-tailed *t*-test and a paired two-tailed *t*-test were used to analyse viability and survival fraction data. Data, except Fig. 1B, are average ± standard deviation.

## 3 Results

### 3.1 Radiolabelling

The labelling efficiencies for THP-trastuzumab labelling with [^67^Ga] Ga-citrate were 100%, 84% and 33% for 25 μL (1.2 MBq), 100 μL (4.9 MBq), and 200 μL (9.7 MBq), respectively. This led to low specific activities of between 0.01 and 0.04 MBq/μg (2–6 GBq/μmol). However, radiolabelling THP-trastuzumab with [^67^Ga]GaCl_3_ achieved >90% radiochemical yield and a specific activity of 0.52 ± 0.16 MBq/μg (78 GBq/μmol). [^67^Ga]GaCl_3_ was therefore used for all further radiolabellings. [^111^In]In-DOTA-trastuzumab gave a radiolabelling yield of 98% with specific activity of 0.61 ± 0.11 MBq/μg (92 GBq/μmol).

### 3.2 Binding and internalisation assay

Both [^67^Ga]Ga-THP-trastuzumab (0.52 ± 0.16 MBq/μg) and [^111^In] In-DOTA-trastuzumab (0.61 ± 0.11 MBq/μg) specifically bound to HER2-positive HCC1954 cells. At 4 nM total antibody concentration, [^67^Ga]Ga-THP-trastuzumab showed higher binding (10.7 ± 1.3%) than [^111^In]In-DOTA-trastuzumab (6.2 ± 1.6%; *p* = .02; 50 × 10^3^ cells, Fig. 1A). The percentage binding of both preparations decreased with increasing trastuzumab concentration. On increasing total antibody concentration to 100 nM, binding of [^67^Ga]Ga-THP-trastuzumab and [^111^In]In-DOTA-trastuzumab was inhibited and reduced to 1.2 ± 1.0% and 0.8 ± 0.9% (p = 0.85), respectively, indicating target-specific binding. However, the total activity bound to cells was higher at the highest antibody concentrations (Fig. 1B). Binding of antibody-free [^67^Ga]Ga-THP and [^111^In]In-DOTA to HCC1954 cells was minimal (0.07 ± 0.02% and 0.03 ± 0.00%, respectively). Binding of [^67^Ga]Ga-THP-trastuzumab and [^111^In]In-DOTA-trastuzumab to HER2-negative MDA-MB-231 cells was 0.18 ± 0.10% and 0.21 ± 0.23%, respectively, again confirming that binding was HER2-specific.

Binding of 4 nM [^67^Ga]Ga-THP-trastuzumab or [^111^In]In-DOTA-trastuzumab to 1 × 10^6^ HCC1954 cells (19.71 ± 1.32% and 11.93 ± 3.32%, respectively) was higher than to 5 × 10^4^ cells (10.7 ± 1.3% and 6.2 ± 1.6%, respectively). Of the cell-bound fraction, 62.1 ± 1.4% and 60.8 ± 15.5% was internalised for [^67^Ga]Ga-THP-trastuzumab and [^111^In]In-DOTA-trastuzumab, respectively.

## 4 Viability (trypan blue staining)

**Fig. 2:**
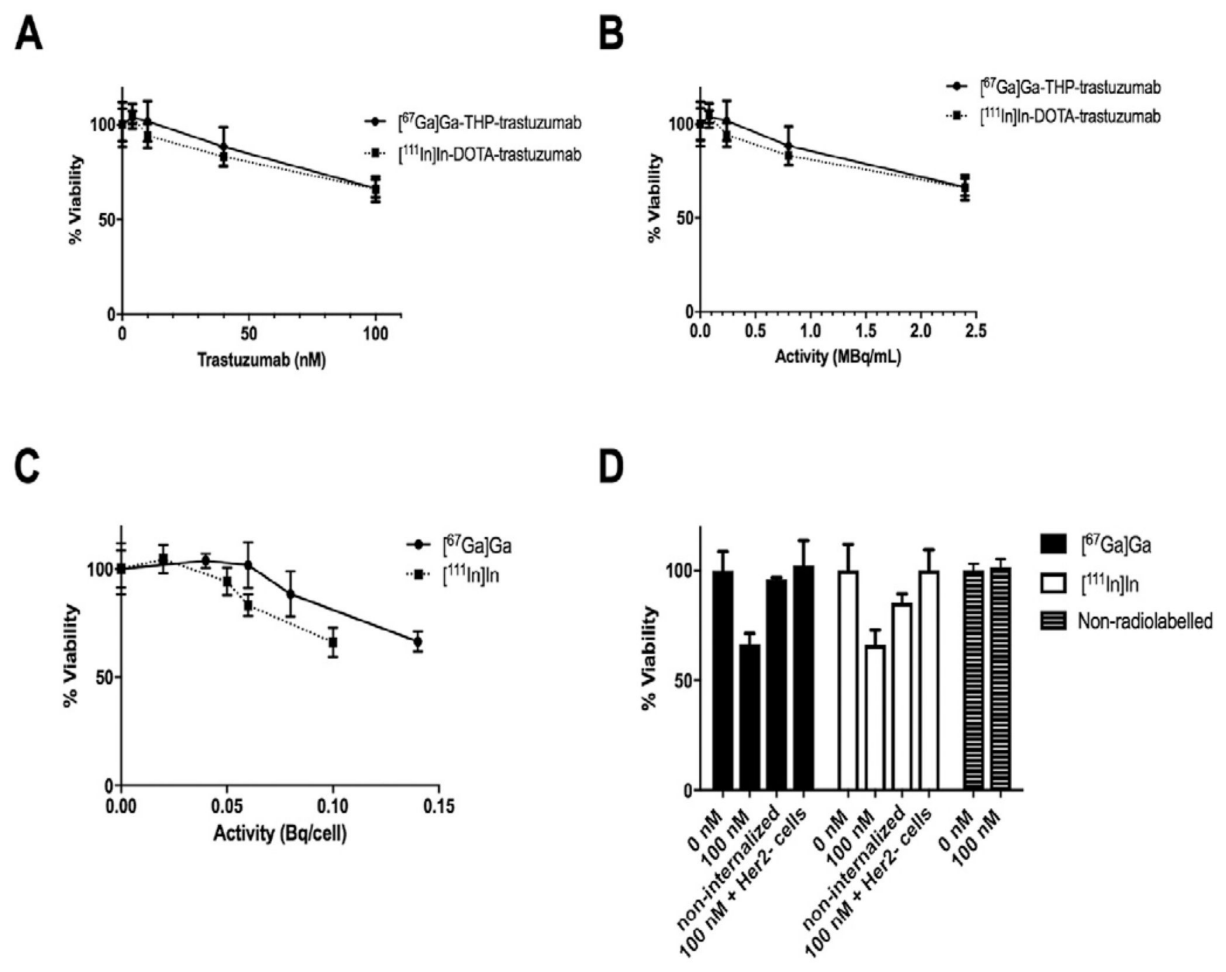
Viability assay (n = 3) using trypan blue 3 days after a one-hour incubation of [^67^Ga]Ga-THP-trastuzumab or [^111^In]In-DOTA-trastuzumab with HER2-positive HCC1954 cells at 0.18 MBq/μg over a range of concentrations (A), activity per mL during incubation (B), and activity bound per cell (C). Controls for the viability assay in D are non-internalised activity in HER2-positive HCC1954 cells incubated with ([^67^Ga]Ga-THP and [^111^In]In-DOTA; 0.5 MBq, 2 MBq/mL), HER2-negative MDA-MB-231 cells incubated with 100 nM radiolabelled trastuzumab, and HER2-positive HCC1954 cells incubated with 100 nM non-radiolabelled trastuzumab (n = 6).

Treatment of HER2-positive HCC1954 cells with 100 nM non-radiolabelled trastuzumab did not affect viability (relative viability 101 ± 3.7% compared to untreated control cells whose relative viability was defined as 100%). Following treatment of HER2-positive HCC1954 cells with either [^67^Ga]Ga-THP-trastuzumab or [^111^In]In-DOTA-trastuzumab, their viability, as measured with trypan blue staining, decreased as the [^67^Ga]Ga-THP-trastuzumab concentration and the activity added to the medium (and hence the cell-bound activity per cell) increased (Fig. 2A, B). Treatment of HCC1954 cells with [^67^Ga]Ga-THP-trastuzumab at 100 nM (2 MBq/mL) gave an average cellular radioactivity of 0.14 Bq/cell and produced significant reduction in cell viability (to 66.5 ± 4.8% of the control value which was defined as 100 ± 8.6% at 0 nM antibody concentration, p = 0.007) (Fig. 2A–C). Under the same conditions, [^111^In]In-DOTA-trastuzumab, gave an average cellular uptake of 0.10 Bq/cell, and reduced viability to 66.2 ± 6.7% of the control value. Thus, the effect of the two radiopharmaceuticals (at similar average cell-bound activity per cell) on viability was not significantly different (p > 0.9). Treatment of HER2-positive cells with non-antibody-conjugated [^67^Ga]Ga-THP (which did not bind to cells) did not measurably reduce cell viability, while non-antibody-conjugated [^111^In]In-DOTA (which also was not cell-bound) marginally decreased cell viability to 85.2 ± 4.0% of the control (Fig. 2D). Thus, both radionuclides were significantly less toxic to HER2-positive HCC1954 cells when not cell-bound (p = 0.0003 for ^67^Ga and p = 0.0146 for ^111^In). The difference in toxicity of non-cell-bound ^67^Ga and ^111^In was not significant (p = 0.09). The results suggest that ^67^Ga and ^111^In need to be bound to cells to achieve significant reduction in cell viability.

### 4.1 Clonogenicity

**Fig. 3:**
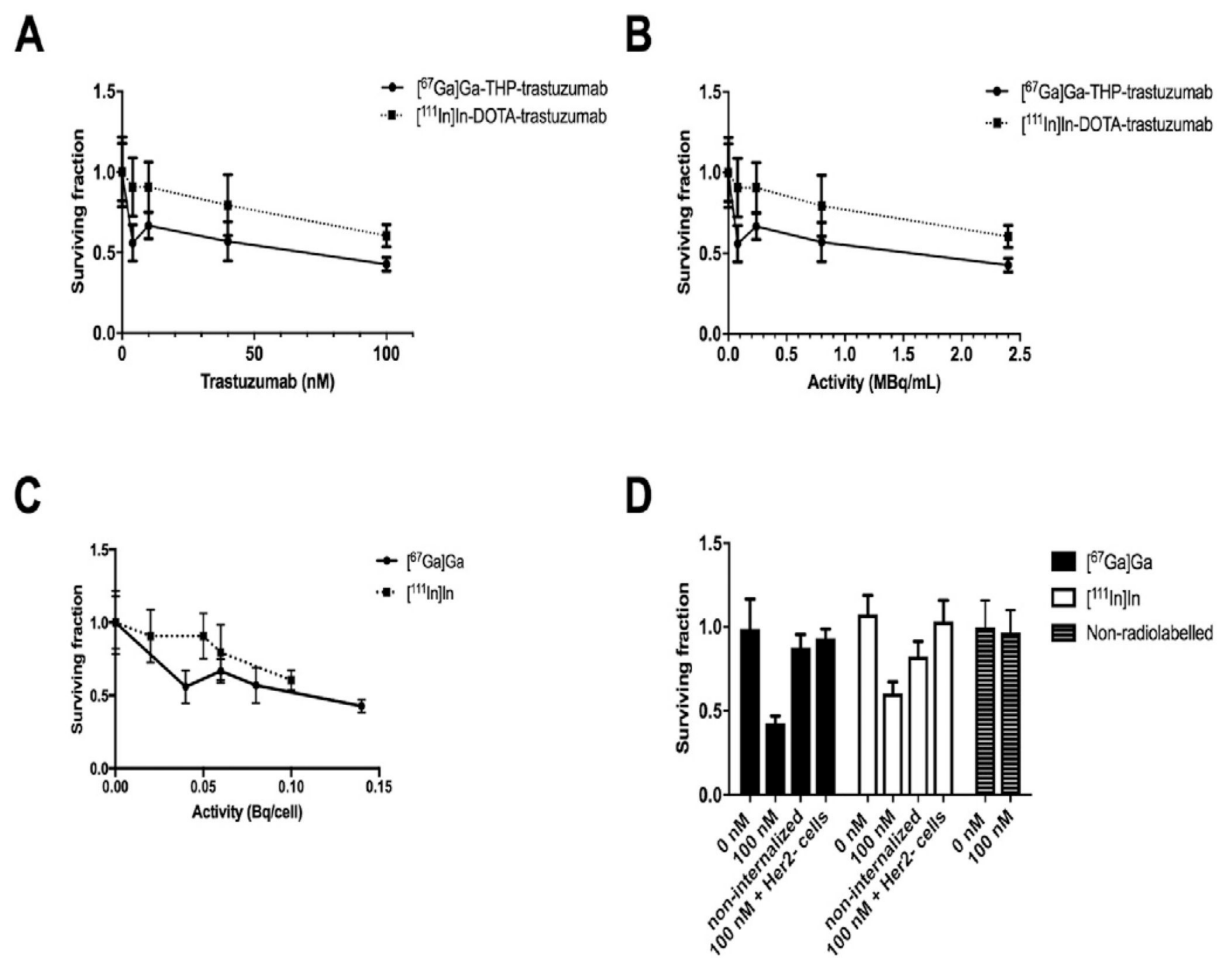
Clonogenic assay (n = 3) of HER2-positive HCC1954 cells incubated for 1 h with [^67^Ga]Ga-THP-trastuzumab or [^111^In]In-DOTA-trastuzumab at 0.18 MBq/μg over a range of concentrations (A), activity per mL during incubation (B), and activity bound per cell (C). In D, controls non-internalised activity in HER2-positive HCC1954 cells incubated ([^67^Ga]Ga-THP and [^111^In]In-DOTA; 1 MBq, 2 MBq/mL), HER2-negative MDA-MB-231 cells incubated with 100 nM radiolabelled trastuzumab, and HER2-positive HCC1954 cells incubated with 100 nM non-radiolabelled trastuzumab (n = 6).

Treatment of HER2-positive HCC1954 cells with 100 nM non-radiolabelled trastuzumab did not affect clonogenicity (relative clonogenicity 0.97 ± 0.13 compared to untreated control cells whose relative clonogenicity was defined as 1.0). Treatment with either [^67^Ga]Ga-THP-trastuzumab or [^111^In]In-DOTA-trastuzumab decreased their relative clonogenicity as concentration of radiolabelled trastuzumab, and consequently the amount of activity per cell, increased (Fig. 3A–C). Treatment of HCC1954 cells with 100 nM [^67^Ga] Ga-THP-trastuzumab and [^111^In]In-DOTA-trastuzumab reduced the clonogenic fraction to 0.43 ± 0.04 and 0.60 ± 0.07, respectively, compared to the untreated control (1.0 ± 0.18; p = 0.002 and 0.01, respectively). The effect of [^67^Ga]Ga on clonogenicity, at similar average activity per cell, was significantly greater than that of ^111^In (p = 0.01). Treatment of HER2-positive cells with non-antibody-conjugated (and hence non-internalised) [^67^Ga]Ga-THP or [^111^In]In-DOTA reduced clonogenicity only marginally (0.88 ± 0.08, p = 0.13 and 0.82 ± 0.09, p = 0.04, respectively) compared to untreated control HCC1954 cells (Fig. 3D). The difference between clonogenic fraction of cells treated with 100 nM [^67^Ga]Ga-THP-trastuzumab (0.43 ± 0.04) and [^111^In]In-DOTA-trastuzumab and (0.60 ± 0.07) and non-cell-bound radioactivity (0.88 ± 0.08 and 0.82 ± 0.09 for [^67^Ga]Ga and [^111^In]In, respectively) was also significant (p = .01 and 0.05, respectively). Clonogenic survival was significantly impaired by treatment with [^67^Ga]Ga-THP-trastuzumab even at very low average activity per cell (<0.05 Bq/cell) but the effect of increasing the activity per cell beyond this level was less dramatic additional effect (Fig. 3B, C).

## 5 Microautoradiography

**Fig. 4:**
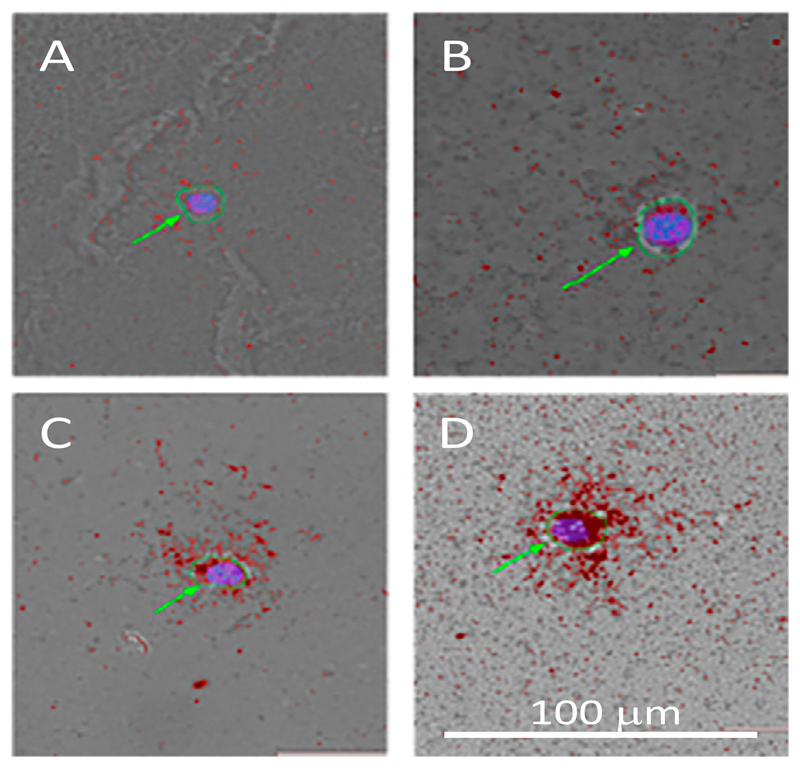
Examples of microautoradiography of HCC1954 cells incubated with 4 nM ^67^Ga-THP-trastuzumab showing mostly highly variable activity per cell ranging from very low (A) to very high (D). The green arrows show the cell boundaries as determined by light microscopy (green circles) with DAPI staining delineating the nucleus (blue). Silver grains are red in the images. Scale bar is 100 μm.

Of the activity added in this experiment (0.04 MBq, 0.07 MBq/μg, 11 GBq/μmol), 14.86 ± 0.38% of [^67^Ga]Ga-THP-trastuzumab was cell-bound. Microautoradiography revealed that radioactivity attached to individual cells varied considerably among the cell population; 15% of cells were classified as unlabelled (≤1 silver grain per cell, e.g. Fig. 4A), 40% showed low levels of labelling (≤10 silver grains per cell, e.g. Fig. 4B), 25% medium labelling (41–90 silver grains per cell, e.g. Fig. 4C) and 20% high labelling (≥91 per cell, e.g. Fig. 4D).

## 6 Discussion

This study extends our previous cell-free system studies using pBR322 plasmids and cells non-specifically labelled using [^67^Ga]Gaoxine, which showed that ^67^Ga causes more DNA damage, and a greater decrease in cancer cell clonogenicity when bound to cells, than the same activity of ^111^In [34]. Here, we have exploited the targeted delivery of radionuclides to HER2-positive cells using the antibody trastuzumab to investigate the selectivity and cytoxicity of targeted radionuclide therapy with ^67^Ga compared to ^111^In [10,42–44].

Initial binding studies were carried out to determine the selectivity of uptake and the concentrations of radiolabelled antibodies required to achieve sufficient uptake of radionuclide in cells to affect measurable impact on cell viability and clonogenicity.

Due to difficulty experienced in radiolabelling trastuzumab with ^67^Ga to a sufficiently high specific activity (i.e. comparable to that achievable with ^111^In) under mild conditions using the DOTA chelators, we used the gallium-specific chelator THP, which can be radiolabelled with gallium radionuclides in under 5 min, at pH 6.5 and room temperature [23] allowing mild and efficient radiolabelling of proteins [33]. Similar specific activities of 0.52–0.61 MBq/μg (78–92 GBq/μmol) could thus be achieved for both [^67^Ga]Ga-THP-trastuzumab and [^111^In] In-DOTA-trastuzumab.

At both low and high concentrations of radiolabelled trastuzumab, the percent binding of [^67^Ga]Ga-THP-trastuzumab to HER2-positive HCC1954 cells was somewhat higher (about 1.5 to 1.7-fold) than that of [^111^In]In-DOTA-trastuzumab. The choice of radionuclide did not affect the fraction of cell-bound radioactivity that was internalised within the cell (as opposed to bound to the cell surface), with over 60% of cell-bound radioactivity being internalised in both cases.

Viability studies using trypan blue staining showed that ^67^Ga induced significant toxicity, but only if incorporated into the cell; at similar concentrations in the media, non-antibody-conjugated [^67^Ga]Ga-THP, which did not bind to cells, did not affect cell viability. [^111^In]In-DOTA, which did not bind to cells, diminished cell viability only slightly, possibly due to a higher fraction of radiation dose coming from gamma emissions in the case of ^111^In. This is consistent with our previous observations [34].

Both [^67^Ga]Ga-THP-trastuzumab and [^111^In]In-DOTA-trastuzumab at 100 nM (2.5 MBq/mL) decreased clonogenic survival significantly compared to untreated HCC1954 cells and compared to HCC1954 cells incubated with 100 nM non-radiolabelled trastuzumab (Fig. 3), suggesting that the mechanism of cell death is mediated by radiation damage. Interestingly, the trends seen in Fig. 3A–C match what one might expect for a survival curve of tumour cells affected through the bystander response [45]. However, further studies will need to be carried out to determine whether the bystander response is indeed activated by these Auger-emitting radiopharmaceuticals. Our data using 100 nM non-radiolabelled trastuzumab (14 μg/mL, 1 h incubation) are in agreement with previous findings that trastuzumab induces no change in viability of HCC1954 cells even after 7 days of incubation at 15 μg/mL [46].

At similar radioactivity concentrations in the medium, [^67^Ga]Ga-THP-trastuzumab is 1.4-fold more toxic (measured as effect on clonogenicity) than [^111^In]In-DOTA-trastuzumab (Fig. 3, A and B). It is likely that only part of this apparent difference in toxicity is associated with the 1.5–1.75-fold more activity of [^67^Ga]Ga-THP-trastuzumab taken up per cell, because when clonogenicity is plotted against the measured average activity per cell (Fig. 3, C), ^67^Ga still shows significantly greater suppression of clonogenicity than ^111^In.

The differences in toxic effects as measured by viability (trypan blue) and clonogenicity indicate that viability and clonogenic assays measure different aspects of cytotoxicity and are complementary rather than alternative methods.

The modest difference in toxicological profile between the two radioimmunoconjugates could originate from differences in the cellular trafficking, or from differences in the electron and photon emission spectra of the two radionuclides. The ^67^Ga conjugate shows greater apparent affinity and uptake than the ^111^In conjugate, and although this does not cause any significant difference in the measured internalised fraction, it implies that differences in subsequent intracellular trafficking, including to the cell nucleus, cannot be excluded. Electron emissions (Auger and Coster-Kronig electrons) of ^67^Ga and ^111^In are similar in total energy (6.24 MeV and 6.75 MeV, respectively) and both also emit internal conversion electrons (0.32 and 0.16 per decay respectively, with a total energy of 28.078 KeV and 25.957 KeV, respectively). However, the energy distribution, and hence range in tissue, of the Auger and Coster-Kronig electrons differ markedly between the two radionuclides. The Supplementary information provides relevant data in the form of plots of yield of electrons per decay against the logarithm of the range [47]. The total energy of the Auger and Coster-Kronig emissions of ^67^Ga is distributed among fewer electrons than is the case for ^111^In (4.7 and 14.7, respectively [47]) and consequently, electrons from ^67^Ga have higher energy per electron, and hence longer range. Fewer Auger electrons emissions per decay than 14.7 [47] have been estimated for ^111^In, namely between 5.84 and 7.43, however these values are still higher than for ^67^Ga (between 4.56 and 4.96) [48]. ^67^Ga emits no electrons with a range below 1 nm, whereas ^111^In emits 8; and ^67^Ga emits around 0.6 electrons per decay with a range encompassing multiple compartments of the cell (1–10 μm), whereas ^111^In emits none. Thus, ^111^In decay is likely to be biologically effective over a much smaller range (<1 μm) than ^67^Ga decay, and the biological effects of decay of ^111^In are likely to be much more dependent on its sub-cellular location than those of ^67^Ga.

Although DNA damage is known to be an important mechanism by which radionuclide decay induces cell killing, it is a currently open and actively investigated question whether the cell nucleus and DNA is the most important target for radionuclide emission, or whether other organelles such as the mitochondria or cell membrane [49,50] are also important radiosensitive targets. Although not explored here, it is known that [^111^In]In-trastuzumab can localise to the nucleus [42,44]. The extent to which this happens is likely to be an important factor in the therapeutic efficacy of the radiopharmaceutical, considering the very short range of the majority of its electron emissions. Nuclear localisation peptide sequences have often been used to further increase therapeutic efficacies of Auger-emitting radiopharmaceuticals [42–44], including some labelled with ^67^Ga [51]. Although other non-nuclear-associated pathways are known that can influence therapeutic efficacy [50], and despite the longer range of the ^67^Ga electron emissions, it is possible that an approach using nuclear localisation sequences will also be of benefit for [^67^Ga]Ga-trastuzumab. The data presented here, however, do not provide insight into the mechanism of cell killing or sub-cellular target of these radioimmunoconjugates.

Microautoradiography demonstrates a wide range of [^67^Ga]Ga-THP-trastuzumab binding among HCC1954 cells, despite the presumption that the cells are a clonally identical population. In fact, 20% of cells in the highest activity group received around a hundred times higher radiation dose than the 15% in the lowest activity group, and a large sub-population of the cells would have received negligible radiation dose. This may account for the observation (Fig. 3B, C) that the surviving cell fraction does not greatly diminish below 0.5 as activity of [^67^Ga]Ga-THP-trastuzumab per cell increases above 0.05 Bq/cell. It implies that the average activity per cell is likely to have very limited utility as a predictor of toxicity. Heterogeneity of target expression is likely to be even greater in cell populations within patients' tumours. This is likely to be the overriding limitation in targeted Auger emitter therapy. The impact of non-homogeneous radiopharmaceutical uptake in limiting anti-tumour effectiveness will need to be considered for any radiopharmaceutical undergoing in vitro and in vivo evaluation and requires further investigation if the antitumour effects of targeted Auger electron-emitting radionuclides are to be better understood.

## 7 Conclusion

[^67^Ga]Ga-THP-trastuzumab specifically reduced cell viability and clonogenicity of HER2-expressing cells, confirming the potential of ^67^Ga as a therapeutic radionuclide as part of a targeted radiopharmaceutical. Diminished viability and clonogenicity only occurred when radioactivity was directly bound to cells and was not caused by radioactivity in the medium. Highly heterogeneous cellular uptake may compromise the efficacy of Auger electron irradiation and the interpretation of data derived from average cellular radioactivity. This merits further investigation using methods, such as microautoradiography, capable of measuring the radioactivity of individual cells.

## Supplementary Material

Supplementary data to this article can be found online at https://doi.org/10.1016/j.nucmedbio.2019.12.004.

Supplementary Information

## References

[1] Mittra ES (2018). Neuroendocrine tumor therapy: (177)Lu-DOTATATE. AJR Am J Roentgenol.

[2] Parker C, Nilsson S, Heinrich D, Helle SI, O’Sullivan JM, Fossa SD (2013). Alpha emitter radium-223 and survival in metastatic prostate cancer. N Engl J Med.

[3] Luster M, Pfestroff A, Hanscheid H, Verburg FA (2017). Radioiodine therapy. Semin Nucl Med.

[4] Gill MR, Falzone N, Du Y, Vallis KA (2017). Targeted radionuclide therapy in combined-modality regimens. Lancet Oncol.

[5] Rathke H, Giesel FL, Flechsig P, Kopka K, Mier W, Hohenfellner M (2018). Repeated (177)Lu-labeled PSMA-617 radioligand therapy using treatment activities of up to 9.3 GBq. J Nucl Med.

[6] Allen BJ (2017). A comparative evaluation of Ac225 vs Bi213 as therapeutic radioisotopes for targeted alpha therapy for cancer. Australas Phys Eng Sci Med.

[7] Heyerdahl H, Abbas N, Sponheim K, Mollatt C, Bruland O, Dahle J (2013). Targeted alpha therapy with 227Th-trastuzumab of intraperitoneal ovarian cancer in nude mice. Curr Radiopharm.

[8] Kiess AP, Minn I, Vaidyanathan G, Hobbs RF, Josefsson A, Shen C (2016). (2S)-2-(3-(1-Carboxy-5-(4-211At-astatobenzamido)pentyl)ureido)-pentanedioic acid for PSMA-targeted alpha-particle radiopharmaceutical therapy. J Nucl Med.

[9] Aghevlian S, Boyle AJ, Reilly RM (2017). Radioimmunotherapy of cancer with high linear energy transfer (LET) radiation delivered by radionuclides emitting alpha-particles or Auger electrons. Adv Drug Deliv Rev.

[10] Falzone N, Lee BQ, Able S, Malcolm J, Terry S, Alayed Y (2018). Targeting micrometastases: the effect of heterogeneous radionuclide distribution on tumor control probability. J Nucl Med.

[11] de Boer M, van Deurzen CH, van Dijck JA, Borm GF, van Diest PJ, Adang EM (2009). Micrometastases or isolated tumor cells and the outcome of breast cancer. N Engl J Med.

[12] Fujisawa M, Miyake H (2008). Significance of micrometastases in prostate cancer. Surg Oncol.

[13] Vallis KA, Reilly RM, Scollard D, Merante P, Brade A, Velauthapillai S (2014). Phase I trial to evaluate the tumor and normal tissue uptake, radiation dosimetry and safety of (111)In-DTPA-human epidermal growth factor in patients with metastatic EGFR-positive breast cancer. Am J Nucl Med Mol Imaging.

[14] De Jong M, Breeman WA, Bernard HF, Kooij PP, Slooter GD, Van Eijck CH (1999). Therapy of neuroendocrine tumors with radiolabeled somatostatin-analogues. Q J Nucl Med.

[15] Seabold JE, Palestro CJ, Brown ML, Datz FL, Forstrom LA, Greenspan BS (1997). Procedure guideline for gallium scintigraphy in inflammation. Society of Nuclear Medicine. J Nucl Med.

[16] Bar-Shalom R, Yefremov N, Guralnik L, Keidar Z, Engel A, Nitecki S (2006). SPECT/CT using 67Ga and 111In-labeled leukocyte scintigraphy for diagnosis of infection. J Nucl Med.

[17] Govindan SV, Goldenberg DM, Elsamra SE, Griffiths GL, Ong GL, Brechbiel MW (2000). Radionuclides linked to a CD74 antibody as therapeutic agents for B-cell lymphoma: comparison of Auger electron emitters with beta-particle emitters. J Nucl Med.

[18] Jonkhoff AR, Huijgens PC, Versteegh RT, van Dieren EB, Ossenkoppele GJ, Martens HJ (1993). Gallium-67 radiotoxicity in human U937 lymphoma cells. Br J Cancer.

[19] Jonkhoff AR, Huijgens PC, Versteegh RT, van Lingen A, Ossenkoppele GJ, Drager AM (1995). Radiotoxicity of 67-gallium on myeloid leukemic blasts. Leuk Res.

[20] Jonkhoff AR, Plaizier MA, Ossenkoppele GJ, Teule GJ, Huijgens PC (1995). High-dose gallium-67 therapy in patients with relapsed acute leukaemia: a feasibility study. Br J Cancer.

[21] Michel RB, Brechbiel MW, Mattes MJ (2003). A comparison of 4 radionuclides conjugated to antibodies for single-cell kill. J Nucl Med.

[22] Ochakovskaya R, Osorio L, Goldenberg DM, Mattes MJ (2001). Therapy of disseminated B-cell lymphoma xenografts in severe combined immunodeficient mice with an anti-CD74 antibody conjugated with (111)indium, (67)gallium, or (90)yttrium. Clin Cancer Res.

[23] Berry DJ, Ma Y, Ballinger JR, Tavare R, Koers A, Sunassee K (2011). Efficient bifunctional gallium-68 chelators for positron emission tomography: tris(hydroxypyridinone) ligands. Chem Commun (Camb).

[24] Knetsch PA, Zhai C, Rangger C, Blatzer M, Haas H, Kaeopookum P (2015). [(68)Ga]FSC-(RGD)_3_ a trimeric RGD peptide for imaging alpha_v_beta_3_ integrin expression based on a novel siderophore derived chelating scaffold-synthesis and evaluation. Nucl Med Biol.

[25] Ma MT, Cullinane C, Waldeck K, Roselt P, Hicks RJ, Blower PJ (2015). Rapid kit-based (68) Ga-labelling and PET imaging with THP-Tyr(3)-octreotate: a preliminary comparison with DOTA-Tyr(3)-octreotate. EJNMMI Res.

[26] Notni J, Pohle K, Wester HJ (2012). Comparative gallium-68 labeling of TRAP-, NOTA-, and DOTA-peptides: practical consequences for the future of gallium-68-PET. Eur J Nucl Med Mol Imaging Res.

[27] Oxboel J, Brandt-Larsen M, Schjoeth-Eskesen C, Myschetzky R, El-Ali HH, Madsen J (2014). Comparison of two new angiogenesis PET tracers ^68^Ga-NODAGA-E[c (RGDyK)]_2_ and (64)cu-NODAGA-E[c(RGDyK)]_2_; in vivo imaging studies in human xenograft tumors. Nucl Med Biol.

[28] Ramogida CF, Cawthray JF, Boros E, Ferreira CL, Patrick BO, Adam MJ (2015). H2CHXdedpa and H4CHXoctapa-chiral acyclic chelating ligands for (67/68)Ga and (111)In radiopharmaceuticals. Inorg Chem.

[29] Simecek J, Notni J, Kapp TG, Kessler H, Wester HJ (2014). Benefits of NOPO as chelator in gallium-68 peptides, exemplified by preclinical characterization of (68)Ga-NOPO-c (RGDfK). Mol Pharm.

[30] Waldron BP, Parker D, Burchardt C, Yufit DS, Zimny M, Roesch F (2013). Structure and stability of hexadentate complexes of ligands based on AAZTA for efficient PET labelling with gallium-68. Chem Commun (Camb).

[31] Hofman MS, Eu P, Jackson P, Hong E, Binns D, Iravani A (2018). Cold kit for prostate-specific membrane antigen (PSMA) PET imaging: phase 1 study of (68)Ga-tris (hydroxypyridinone)-PSMA PET/CT in patients with prostate cancer. J Nucl Med.

[32] Young JD, Abbate V, Imberti C, Meszaros LK, Ma MT, Terry SYA (2017). (68)Ga-THP-PSMA: a PET imaging agent for prostate cancer offering rapid, room-temperature, 1-step kit-based radiolabeling. J Nucl Med.

[33] Nawaz S, Mullen GE, Sunassee K, Bordoloi J, Blower PJ, Ballinger JR (2017). Simple, mild, one-step labelling of proteins with gallium-68 using a tris(hydroxypyridinone) bifunctional chelator: a 68Ga-THP-scFv targeting the prostate-specific membrane antigen. EJNMMI Res.

[34] Othman MF, Mitry NR, Lewington VJ, Blower PJ, Terry SY (2017). Re-assessing gallium-67 as a therapeutic radionuclide. Nucl Med Biol.

[35] Ulaner GA, Hyman DM, Ross DS, Corben A, Chandarlapaty S, Goldfarb S (2016). Detection of HER2-positive metastases in patients with HER2-negative primary breast cancer using 89Zr-trastuzumab PET/CT. J Nucl Med.

[36] Slamon DJ, Clark GM, Wong SG, Levin WJ, Ullrich A, McGuire WL (1987). Human breast cancer: correlation of relapse and survival with amplification of the HER-2/neu oncogene. Science.

[37] Swain SM, Baselga J, Kim SB, Ro J, Semiglazov V, Campone M (2015). Pertuzumab, trastuzumab, and docetaxel in HER2-positive metastatic breast cancer. N Engl J Med.

[38] Amiri-Kordestani L, Blumenthal GM, Xu QC, Zhang L, Tang SW, Ha L (2014). FDA approval: ado-trastuzumab emtansine for the treatment of patients with HER2-positive metastatic breast cancer. Clin Cancer Res.

[39] Scasnar V, van Lier JE (1993). The use of SEP-PAK SI cartridges for the preparation of gallium chloride from the citrate solution. Eur J Nucl Med.

[40] Ma MT, Cullinane C, Imberti C, Baguna Torres J, Terry SY, Roselt P (2016). New tris (hydroxypyridinone) bifunctional chelators containing isothiocyanate groups provide a versatile platform for rapid one-step labeling and PET imaging with (68)Ga (3.). Bioconjug Chem.

[41] Reilly RM, Kiarash R, Cameron RG, Porlier N, Sandhu J, Hill RP (2000). 111In-labeled EGF is selectively radiotoxic to human breast cancer cells overexpressing EGFR. J Nucl Med.

[42] Costantini DL, Bateman K, McLarty K, Vallis KA, Reilly RM (2008). Trastuzumab-resistant breast cancer cells remain sensitive to the auger electron-emitting radiotherapeutic agent 111In-NLS-trastuzumab and are radiosensitized by methotrexate. J Nucl Med.

[43] Costantini DL, McLarty K, Lee H, Done SJ, Vallis KA, Reilly RM (2010). Antitumor effects and normal-tissue toxicity of 111In-nuclear localization sequence-trastuzumab in athymic mice bearing HER-positive human breast cancer xenografts. J Nucl Med.

[44] Li HK, Morokoshi Y, Daino K, Furukawa T, Kamada T, Saga T (2015). Transcriptomic signatures of Auger electron radioimmunotherapy using nuclear targeting (111)intrastuzumab for potential combination therapies. Cancer Biother Radiopharm.

[45] Prise KM, O’Sullivan JM (2009). Radiation-induced bystander signalling in cancer therapy. Nat Rev Cancer.

[46] Tormo E, Adam-Artigues A, Ballester S, Pineda B, Zazo S, Gonzalez-Alonso P (2017). The role of miR-26a and miR-30b in HER2+ breast cancer trastuzumab resistance and regulation of the CCNE2 gene. Sci Rep.

[47] Howell RW (1992). Radiation spectra for Auger-electron emitting radionuclides: report No. 2 of AAPM Nuclear Medicine Task Group No. 6. Med Phys.

[48] Falzone N, Lee BQ, Fernández-Varea JM, Kartsonaki C, Stuchbery AE, Kibédi T (2017). Absorbed dose evaluation of Auger electron-emitting radionuclides: impact of input decay spectra on dose point kernels and S-values. Phys Med Biol.

[49] Kam WW, Banati RB (2013). Effects of ionizing radiation on mitochondria. Free Radic Biol Med.

[50] Paillas S, Ladjohounlou R, Lozza C, Pichard A, Boudousq V, Jarlier M (2016). Localized irradiation of cell membrane by auger electrons is cytotoxic through oxidative stress-mediated nontargeted effects. Antioxid Redox Signal.

[51] Koumarianou E, Slastnikova TA, Pruszynski M, Rosenkranz AA, Vaidyanathan G, Sobolev AS (2014). Radiolabeling and in vitro evaluation of (67)Ga-NOTA-modular nanotransporter—a potential Auger electron emitting EGFR-targeted radiotherapeutic. Nucl Med Biol.

