# Supplementary material for: In vitro cytotoxicity of Auger electron-emitting [67Ga]Ga-trastuzumab
Source: Nucl Med Biol. Author manuscript; Available in PMC 2020 Mar 27. (PMC7099941; doi:10.1016/j.nucmedbio.2019.12.004)
Supplement: Supplementary Information [file EMS86062-supplement-Supplementary_Information.pdf]

Supplementary Information

In vitro cytotoxicity of Auger electron-emitting [^{67}Ga]Ga-trastuzumab

Muhamad Faiz bin Othman, Elise Verger, Ines Costa, Meena Tanapirakgul, Margaret S Cooper, Cinzia Imberti, Valerie J Lewington, Philip J Blower, Samantha YA Terry

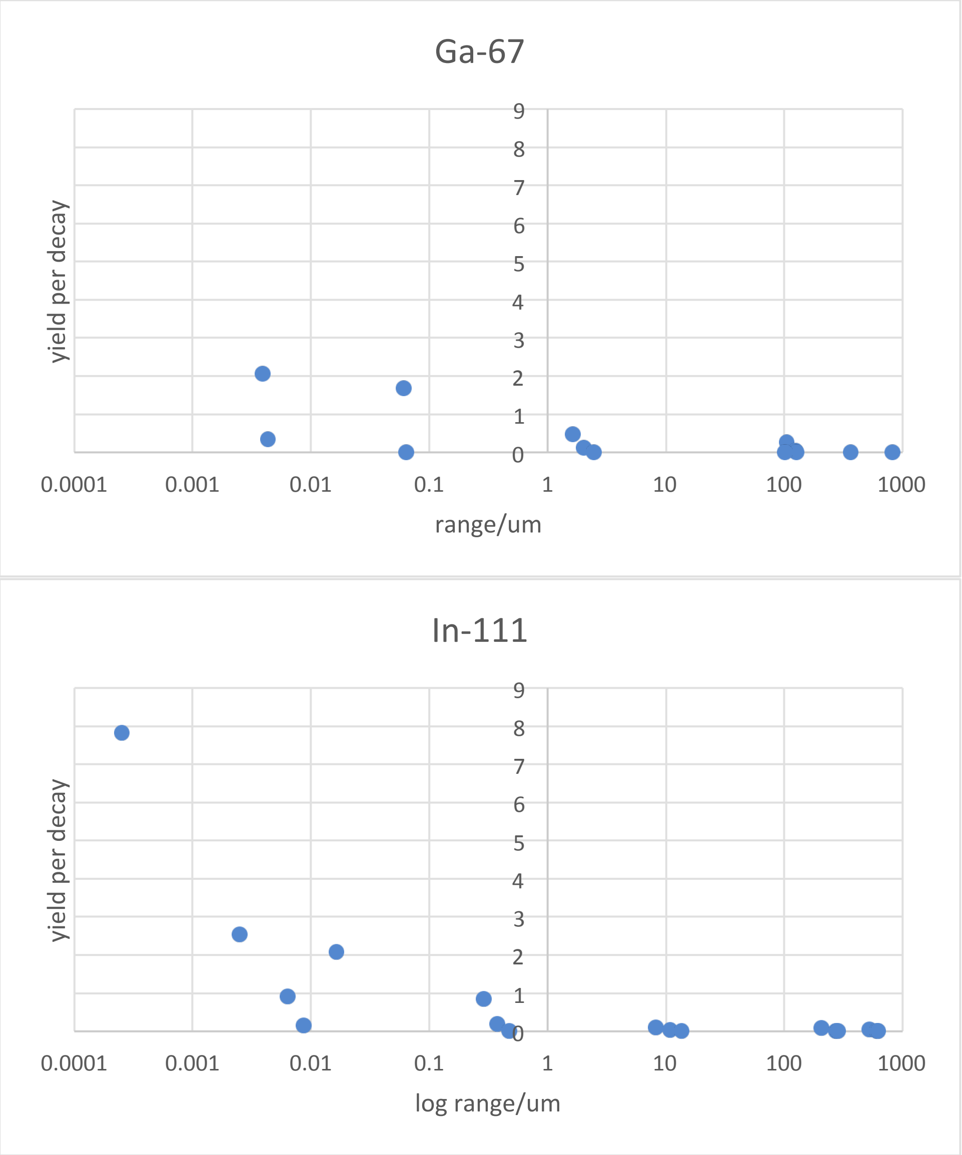

Figure S1. Plots of electron emission abundance versus range in tissue for gallium-67 and indium-111, including Auger electrons, Coster-Kronig electrons and internal conversion electrons. Data taken from Howell RW. Radiation spectra for Auger electron emitting radionuclides: Report No. 2 of AAPM Nuclear Medicine Task Group No. 6. Med Phys 1992;19:1371-1383.
